# Who decides on time? Mad Time as a disruptor of normative research politics and practices

**DOI:** 10.3389/fsoc.2025.1559616

**Published:** 2025-04-17

**Authors:** Aimee Sinclair

**Affiliations:** School of Allied Health, Curtin University, Perth, WA, Australia

**Keywords:** Mad Time, madness, PQI, psychiatrization, methodology

## Abstract

There is an increasing recognition of the epistemic injustice perpetrated against individuals deemed mad, leading to a push for the inclusion of their voices in research and academia. Nevertheless, despite being predominantly enacted as progressive, the inclusion of individuals deemed mad within research practices and spaces often fails to disrupt the ways in which methodology is conceptualized and practiced, contributing to the ongoing psychiatrization and exclusion of Mad practices and, more broadly, failing to produce alternatives to carceral responses to madness. In this article, I consider both the potential for methodology to produce temporal violence as well as the potential of Mad Time to disrupt normative and often sanist research practices. To achieve this, I weave together theorizing on Mad Time, post-qualitative inquiry, the experiences of peer support workers, and my own temporal conflicts in attempting to madden research within academia. I propose three ways in which Mad Time may provoke alternative methodological practices that move us closer to epistemic justice: rethinking the concept of data, embracing stumbling, circling, scrambling (becoming), and valuing variations in pace. I conclude by reflecting on the possible implications that thinking with Mad Time might hold for both research and activism, both within and outside of academia.

## Introduction

*“Sometimes you just want your curtains closed [in a hospital room]…I’m having this sort of memory of the darker days, of both internally, but also wanting a darker room for whatever reason, and then when people come in and just thrust open your blinds and just completely walk into your space because its whatever time on their shift and decide for you that its morning.” (Paula)*

For individuals deemed mad, time is often thrust upon us. For example, in inpatient units, others decide what time of day it is and what we should be doing according to that time. Days are scheduled around others’ timeframes and according to normative expectations of what should happen and when. We are diagnosed according to time; we might fail to get out of bed at the right time, and our minds and bodies move too quickly, slowly, or inconsistently. Time can often feel as if it is standing still: we wait for the doctor, for medication, to be listened to. We wait to be allowed to resume life: our future on hold. At other times, time is rushed by: meetings with the psychiatrist, diagnosis, and discharge happen before we have had time to grasp what such occurrences mean. Our own paces, orientations, and conceptualizations of time are dismissed or devalued.

Such temporal violence, produced through entanglements of psychiatric logic and mental health systems, is similarly produced through methodology. Temporal research orientations and practices often go unremarked, yet shape how, when and where knowledge is produced. They shape the questions researchers ask and how they are answered. They include ideas about where research starts and stops (and thus what constitutes research), how research should progress, and expectations concerning the pace of research. Such temporalities play a large part in the ongoing dominance of psychiatric thinking and the ongoing exclusion and erasure of Mad knowledge and practices within academia and beyond (Russo, 2022). The temporal orientations of academic knowledge production have long excluded those deemed Mad. As Sheppard (2020, p. 39) describes regarding the exclusion of disabled bodyminds more generally, we are “too slow, too fast, too uncontrolled, too reliant, too different, too much and also not enough.” In this article, I use Mad Time to consider the temporal aspects of research that uphold psychiatrization through the exclusion of Mad knowledge and practices.

Furthermore, I consider Mad Time as a potential disruptor to normative research temporalities, inviting us to “imagine and enact methodology otherwise” (Eales and Peers, 2021, p. 164). Rather than conceptualiz madness through pathology and fear, I consider how we might value the ways in which bodyminds^1^ slow down, speed up, ruminate on the past or future, and refuse to do things at the supposedly appropriate time. How might we recognize the important learnings that can come from Mad “moments of rupture and disorientation?” (Davies, 2024, n.p.). How might Mad Time stimulate new ways of enacting methodology that move us toward alternatives to psychiatrization and the abolition of carceral systems and responses to distress?

Whilst entanglements of time, ableism, heteronormativity, and methodology have been explored from crip and queer perspectives (Atkinson et al., 2024; Humphrey et al., 2023; Humphrey and Coleman-Fountain, 2023; Rodgers et al., 2022), explorations focused more specifically on sanist research temporalities and the potentials of Mad Time are limited. Such explorations are important and pressing, given the increased inclusion of lived experience within mental health research, often in ways that continue to psychiatrize and fail to recognize the generative potential of Mad knowledge and practices (Rose and Kalathil, 2019; Landry, 2017; Ross et al., 2023; Russo, 2022; Sinclair et al., 2023a). This epistemic injustice has far-reaching consequences, contributing to ongoing carceral responses to psychiatrized distress.

I start by outlining Mad Time as described in the existing literature, albeit with broad strokes, while acknowledging that explorations of Mad Time, like madness, “should always leave room for different views and stories” (Smith, 2024, np). I then describe the relations that have provoked my thinking about Mad Time, research politics and practices. Such relations, loosely but not wholly contained within a research project examining the politics of inclusion, include temporal conflicts I experienced attempting to madden research and navigate academia as a Mad scholar, theorizing on Mad Time, practices of post-qualitative inquiry, past experiences of peer support workers and their visceral reverberations into the present, and dreaming of Mad futurities.^2^ Using quotes from peer support workers as provocation points, I provide three examples of how methodology can be exclusionary and reinforce psy-knowledge, as well as the ways in which Mad Time may disrupt such practices. I consider the ways in which, as researchers (whether as researchers employed within academia, individuals contributing to research, or those of us doing our own theorizing and activism outside of academia), we may draw on Mad Time to unsettle sanist research practices and deepen our activism.

### Mad Time

In thinking with Mad Time, I am stirred by Cosantino’s (2022, p. 1) powerful poetic meditation on Mad trans time, describing Mad trans time as “a deeply embodied theorizing, challenging and actively disrupting normative temporalities, blurring the artificial boundaries between past, present, and future; knowing and (un)knowing; being and becoming.” Mad Time signifies multiple and diverse ways of thinking, feeling, and doing time that coincide with experiences of madness, the “material differences of life as part of a subaltern group” (Price, 2024), and that sit in tension with sanist conceptualizations of the ‘right’ way of being in/through time. Bruce (2017, p. 1) provides examples of Mad Time: “the quick, restless time of mania; the slow, sorrowful time of depression; the infinite, exigent now of schizophrenia; and the spiralling, zigzagging now-then-now-then of melancholia.” McEwan (2023, p. 35) works toward an obsessive-compulsive Mad Time, describing how such experiences “both speed up time in the frantic repetition of obsession and compulsion” and yet externally “appear slow and illogical.”

Mad Time, like queer and crip time, may involve a refusal to embrace curative futures, time outside of productivity, flexible time, and departures from linear progress, particularly linear narratives of recovery (Kafer, 2013, p. 34; Sheppard, 2020). Mad Time “tears calendars, smashes clocks, ignores calls for timeliness, builds makeshift time machines, writes “poetry from the future” (Bruce, 2021, p. 204). Drawing from their lived experience, Morrigan (2017, p. 56) proposes that “queer temporalities” of “traumatized minds” provide “a creative, flexible and nonlinear way of relating to time,” opening up possibilities for different ways of being in the world, rather than a “problem, a tragedy, or an unfortunate condition requiring a cure.” Thus, Mad Time defies “the Eurocentric, heteronormative, capitalist, rationalist clock-time that reigns in the modern West” (Bruce, 2021, p. 204). It defies normative futures associated with rationalist subjects. It defies, as Paula describes in the opening quote of this article, having to ‘rise and shine’ at a certain time as defined by clinicians within a mental health unit. We madden time whenever we “infuse the disruptive potential of [madness] into normative spaces and interactions” (Price, 2015, p. 269).

Thinking with Mad Time involves centring the temporal expectations and activism of individuals who encounter psychiatric classification and violence. As an aspect of Mad theorizing, Mad Time both critiques and aims to transcend sanism whilst recognizing the intersection of sanism with other forms of oppression (Costa and Ross, 2022; LeFrançois et al., 2016). It invites consideration of the potential of madness to subvert the status quo, disrupting dominant conceptualizations of madness as only ever requiring cure, treatment, or management.

Numerous studies have not been written specifically about Mad Time but have, and continue to be, influential in thinking about Mad Time and knowledge production, and therefore require honoring here. First, the concept of Mad Time is heavily indebted to scholars and activists theorizing on crip, queer, and trans time (Bruce, 2017; Davies, 2024; Kafer, 2013; Price, 2024; Samuels, 2017). These concepts have, in common, a critique of normative assumptions about time and their oppressive effects, a centring of the experiences of individuals and communities that fail to measure up to such ‘normal’ temporalities, as well as provoking alternative ways of being in the world that not only accommodate but value difference. Such experiences are often intimately intertwined, with many theories interweaving them within their analysis. Bruce (2021), for example, writes of both Black and Mad Time, whilst Cosantino (2022) and Morrigan (2017) speak of Mad, trans, and queer time. Kafer (2013) weaves together feminist theorizing with queer and crip time, including both disabled bodies and minds under the banner of crip time.

Whilst recognizing the intimate entanglement of Mad Time with other temporalities, I join others in arguing that Mad Time holds generative potential as a distinct analytical concept (Smith, 2017). In particular, I position Mad Time as overlapping with, but separate from, crip time. Mad Time, as an aspect of Mad studies, communities, and activism, has developed alongside but distinct from crip and disability communities. For example, Mad studies, a discipline that brings together Mad research, theorizing, activism, and Mad culture, has a complex relationship with disability studies (Jones and Kelly, 2015). Whilst emerging partially via critical disability studies, Mad activism and scholarship has tended to occur separately from disability politics given the ambivalence around mainstream disability studies ability to theorize madness, and more critical perspectives within disability scholarship “pushed to the margins” in favor of more pragmatic (and fund-able) research (Cohen, 2017, p. 2; Sapey et al., 2015). Mad Time, as a distinct analytical concept, centers the temporal experiences and activism of individuals who encounter psychiatric classification and violence and draws specific attention and critique to “psy-centred ways of thinking, behaving, relating, and being” in a way that cannot be done via a more generalized crip lens that focuses on ableism (Menzies et al., 2013, p. 13). Similarly, Mad Time may be useful, but cannot speak fully, to the distinct experiences and ways of theorizing and responding within crip and disability communities.

Whilst I argue for the utility of these as distinct concepts, I also recognize the intimate entanglement between not only crip and Mad experiences but also a range of other experiences and that “we-who are not one and the same – are in this troubled world…together” (Braidotti, 2022, p. 241). There remain many similarities not only between experiences but also in the way such experiences are responded to as only ever requiring cure, treatment, or management. As such, there is great benefit in drawing from these concepts when theorizing ways in which normative temporalities may be disrupted within academia and beyond. I continually learn from crip theorizing and crip communities, and I desire to recognize the value of thinking together without “flatten[ing] the diversity of disabled/mad/chronically ill/debilitated communities” (Gauthier-Mamaril, 2024, p. 1). Within this article, I thus quote those who have written on crip time where such theorizing overlaps, or may speak to, Mad experiences, thinking, or practices.

Furthermore, it is imperative that thinking with Mad Time involves a consideration of First Nation knowledge and practices of resistance to the ongoing violence of both colonial and sanist temporalities. First Nation peoples from colonially named ‘Australia’ have orientations to time that differ from colonial time and temporalities (Yunkaporta, 2023). Indigenous scholars (activists, poets, artists, teachers, and elders) have highlighted how such orientations enable meaningful engagement and sustainable relationships, disrupting colonial academic temporalities (Wright et al., 2016). Writing as a wadjela (white person/colonial settler) on the stolen lands of the Wadjuck Noongar people, I reference such knowledge tentatively, given that they are not culturally bound to me, and the risk of appropriation within academia is significant. Indigenous worldviews and practices are often sidelined through academic claims of new ontologies and practices, despite Indigenous theorizing having always acknowledged complex and ever-shifting entanglements of the social and material (Arnold et al., 2021; Milroy, 2021; Price, 2024; Todd, 2016).

Mad Time as an analytical concept is also indebted to activists and scholars who have theorized and practiced alternative ways of thinking and doing within Mad communities (Beresford and Russo, 2021; LeFrançois et al., 2013; Russo and Sweeney, 2016).^3^ My own thinking about Mad Time would not be possible without peers: individuals with lived experience of psy-oppression and/or misfitting with normative temporalities who have shared their experiences and theorized alongside me, both within the scope of a formal research project (which I discuss shortly) as well as more broadly within the community. Whilst many did not use the language of Mad Time, their sharing of experiences becoming entangled with, and resisting, normative temporalities and understandings of such have provoked my own theorizing and maddening of time. Mad individuals, particularly those outside academia, are rarely considered theoretical provocateurs and critical theorists. Nevertheless, those who draw on madness in their thinking and doing always have subversive potential.

Lastly, in considering the generative potential of Mad Time, I resist romanticizing madness or Mad Time. As highlighted by Bruce (2021), madness, both one’s internal experiences as well as the experiences of being medicalized and discriminated against, can be both a source of pain and a resource for revolution. Samuels (2017) makes the same argument about crip time, describing how, in their life, it has operated as a form of liberation as well as a site of loss and alienation. There are “risks that haunt these temporalities. Manic time might rush recklessly into danger; depressive time might become so deeply wedged in woe that it does not ever get free; schizophrenic time might be crushed between history’s hurt and the future’s threat; melancholic time might collapse under the weight of the lost and dead that it carries” (Bruce, 2021, p. 229). Considering Mad Time means acknowledging its potential for both pain and revolution.

## Methodology/anti-methodology

My thinking for this article is contained somewhat within the scope of a formal research project exploring the politics of inclusion for peer support within mental health systems, which I describe here. However, as I argue in this article, Mad Time encourages consideration of how the boundaries we draw around a research project are artificial, with researcher desires, experiences and histories, technology, material environments, discourses and so on, seeping in and out of the research assemblage, affecting and being affected by the knowledge produced. By assemblage, I refer to relations of socio-material elements (bodies, meanings, emotions, objects, places, and technologies) organized and held together temporarily that produce knowledge: subjects, objects, and concepts in particular ways. The notion of a research assemblage reflects the complex and ever-shifting entanglements (co-researchers, contributors, consent forms, technology, methods, ethics committees, time, desires, and theories, among others) that produce knowledge (Bettez, 2015; Ellingson and Sotirin, 2020).

Time was not the focus of this research project. Instead, the project sought to explore the effects of peer support becoming increasingly entangled with mental health systems, defined and operationalized as a formal occupation. The research involved several practices of inquiry: thinking with Mad and post-humanist theory, discussions with peer support workers, analysis of policy documents and research practices (Bacchi and Goodwin, 2016), collaborative and solo practices of coding with wonder (Mac Lure, 2013), mapping (Martin and Kamberelis, 2013), and experimenting with afflexivity (Setchell et al., 2021). I conceptualize these as inquiry practices, as I did not follow rigid protocols nor systematically apply a technique or procedure to produce knowledge, as implied by the concepts of ‘method’ and ‘methodology’ (Koro-Ljungberg, 2016). Rather, I sought to think with Mad and post-humanist theories and concepts alongside others with experience navigating mental health systems. The research aimed to not represent inclusion but rather deconstruct it, to agitate and provoke alternative ways of thinking about inclusion, peer support, and madness in ways that may ultimately change how madness is responded to.

Unlike conventional methodology, Mad thinking and doing is far from systematic or replicable. Whilst methodology seeks to structure, fix and contain, madness invites us to be unruly, to disrupt, to refuse, and to dream otherwise (Bruce, 2021; LeFrancois and Voronka, 2022; Smith, 2024). Madness as methodology involves refusing to be loyal to systemic or fixed research methods at the expense of generating alternative knowledge and practices. It means adopting an anti-methodological stance, challenging conventional boundaries of method and turning instead toward the “unmanageable, incredible, illegitimate” (Smith, 2017, p. 1). However, all research studies, particularly that situated within academia, follow some sort of established path. Our work is captured by dominant forces, even if it does veer off in all sorts of illegitimate and wonderful directions that disrupt the status quo at various points. Thus, I describe partly methodology and partly madness as “anti-methodology” (Smith, 2017, p. 1).

A total of 15 individuals who had experience providing peer support within mental health systems in a formal capacity contributed to the research via one-on-one discussions. Such discussions might be referred to in more conventional qualitative research as dialogic interviews. However, as I will go on to discuss shortly, I resist such language that suggests a researcher gathers ‘data’ from/on contributors. Rather, my desire, influenced by the values of both peer support (Stratford et al., 2019) and survivor research (Faulkner, 2004; Landry, 2017), was for us to share our experiences and practices as peer support workers within the (Australian) mental health system, “actively engaged in the creation of knowledge” together (Motta, 2016, p. 42). Given the nature of peer support roles as involving the drawing on one’s own lived experiences of distress and/or navigating psy-systems, our conversations often traversed our experiences as both ‘service user’ and ‘worker’ within mental health systems. I do not conceptualize the experiences shared in these discussions as ‘representative’ of peer support worker experiences but rather as providing provocations to think and feel “otherwise” (Taguchi, 2012, p. 272). Our conversations were held in person, over the phone or online, lasting 1–2 h.

After these conversations, I initially mapped the relations in which peer support work becomes entangled, plugging in policy and research analysis, Mad and post-humanist theorizing, with that shared in our discussions. Contributors were then invited to come together to continue this mapping of the affective relations that peer support workers become entangled with through inclusion (see Sinclair et al. 2023b; Sinclair and Mahboub, 2024 for further detail). Four individuals who had contributed via the initial one-on-one discussions joined this workshop, alongside myself and an additional lived experience academic. Together, we used prompts to map potential relations that produce dominant ways of thinking and responding to distress and those that provoke alternative possibilities.

Whilst time was not the focus of the research, it was an affective element that continually surfaced throughout our discussions and became an important part of my thinking about the effects of inclusion (see Sinclair, in press). As I thought with/through such temporal aspects, I also increasingly reflected on my own journeying through academia, both as a Mad scholar misfitting academic temporalities (Price, 2024; Rodgers et al., 2022) and my attempts at research aligned with Mad anti/methodologies and survivor research within sanist academic entanglements (Faulkner, 2004; Landry, 2017; LeFrancois and Voronka, 2022; Smith, 2024). From the beginning of my research journey, normative temporalities had clashed with my desires to value survivor research, Mad ontologies, and my own Mad ways of thinking, feeling, and existing. For example, I struggled to formulate a fixed and linear methodological plan for ethics approval without having spoken to contributors about the different ways they may desire to undertake the research and knowing this may change over time as we create knowledge together (see Sinclair and Ridley, 2022). However, without the language or framings to conceptualize such, these tensions largely went unspoken, pushed within as something potentially wrong with me. As Davies (2024; n.p.) highlights that individuals deemed mad, such as myself, are taught to “distrust their/our own gut reactions, feelings, sensations, and emotions, and to even deem our feelings untrue, unrealistic, or false through mental health interventions.”

It was not until such affects became entangled with thinking alongside fellow peer workers and others who have theorized on temporalities that I started to make sense of such tensions. Moments where peer support workers spoke of time intensified my thinking, making me pause, whilst at the same time, connections were fired up as we recalled and shared incidents, details, and feelings. I also noticed temporal tensions between what was left unsaid and what was assumed within incidents or practices. Temporalities as a concept became an important part of my thinking about the effects of peer support inclusion (Sinclair, in press). However, more relevant to this article, this thinking and feeling extended to my reflections on methodology and the research journey itself. I became increasingly aware of the deep entanglement of normative temporalities within research politics and processes and how these sat with/against my own research practices.

Ironically, during this process, my bodymind slowed. I struggled to get out of bed. Tasks took me what seemed like so long, and my brain would not work as quickly as I wanted it to. Everyday conversations and decisions were hard as I struggled to process the information coming in and integrate it with what was already known. I have been to this place many times before. I know with time, it will pass. I work with people close to me to clear the decks – I cancel appointments and work, and we sit tight. We eat frozen meals. I potter around. Spend time in the garden. Go for slow walks. Slowly. Slowly, the world starts to feel safe enough to re-enter, my bodymind feels strong enough to re-engage, and I return to operating at a pace considered normal within academic spaces underpinned by neoliberal, capitalist, colonial, and patriarchal ideals. Doing so requires leaning on my socio-economic privilege, performing as a ‘supercrip’ to catch up on what is considered my reduced productivity (Clare, 2015).

It is easy in such moments for me to curse my bodymind for failing to keep up with normative temporal orientations and for failing to do research in a way that aligns with academic schedules and expectations (Price, 2024; Rodgers et al., 2022). However, readings on Mad, crip, trans, queer, and Indigenous time alongside the knowledge and experiences of peer support workers have enabled a growing part of me to celebrate my bodymind’s resistance to the normalized ideals of productivity within research assemblages and the chrono-normative ordering of research. Rather than seeing bodyminds as needing discipline, I started to use Mad Time to consider such failures as productive, becoming curious about how such thinking might be applied to methodology. How might my own, and others’, Mad experiences of temporal misfitting be considered valuable?

My own experiences form part of what is contained within this article. I share my reflections on both engaging in this research and navigating different temporal orientations and expectations alongside the experiences of others as part of my commitment to becoming mad in our thinking and doing together. Bringing forward and sharing one’s experiences of madness and using such experiences to inform relationships, research, and activism is an integral part of peer work, survivor research, and Mad theorizing. However, drawing on a relational ontology, I am uncomfortable positioning the work as autoethnographic. I do not see myself and my experiences as a separate piece of ‘data’ to be analyzed alongside those of others, nor do I position them representative of a wider cultural experience. Instead, I conceptualize my body, history, emotions, desires, and experiences as deeply entangled with the research, both shaping and being shaped by it. For example, I consider the experiences and knowledge shared within discussions with peer support workers as produced *in relation* to other elements, including bodily responses, ideas, objects (technology, consent forms), expectations (for example, what we thought the other might want to hear), desires (for example, what I/they/we wanted to get off one’s chest), and dominant discourses (shaping what can and cannot be made sense of and articulated).^4^ I thus share my own experiences and reflections, not as representative, but as provocation points to think differently about madness and methodology.

A further element, post-qualitative inquiry (PQI), is also interwoven in this research. Similar to Mad methodologies, PQI rejects pre-existing methods and definitions of how research should be done, arguing that these methods and research protocols serve as exclusionary measures and, too often, simplify complex and multifaceted phenomena. Instead of prioritizing methods, PQI requires a commitment to thinking rigorously with theory and concepts (Koro-Ljungberg, 2016; Lather and Pierre, 2013; Liddiard et al., 2019; Murris, 2020; Pierre, 2021). Taken together, both Mad methodologies and PQI refuse “to make mad subjects both knowable and governable, or to make sense of that which cannot and should not be reduced to the rationalist’s desire for uniformity, consistency, universality and conformity to the dominant logics of the sanestream” (LeFrancois and Voronka, 2022, p. 108). Woven together, these approaches push toward enacting research as performative rather than representative, political rather than ‘objective’, prompting questions such as ‘What do our research practices produce?’ and ‘In what ways might we identify and support relations that produce alternatives to psychiatrization?’

These relations between Mad Time, PQI, peer support workers’ experiences, my own Mad desires, academic processes, and expectations cannot be separated into a set of linear and replicable methodological steps. Rather, they are part of the research assemblage: deeply entangled and collectively, they have produced what the thinking outlined below. I desire this study to be taken as a provocation, to open up ways for thinking about madness and methodologies as diverse and multiple, rather than taking such work as representative of Mad experiences and relations to time or arguing for the ‘right’ way to do research. I have used a variety of inputs to think through and against my own experiences of madness and temporality, including supervision, engaging with affective tensions and differences within transcripts, and thinking with theory across disciplines. However, I will have undoubtedly erased experiences and thinking that do not reflect my own. Madness exists in many forms, with many varied implications. Furthermore, my privilege and positionality in the world have, in many ways, sheltered me from the more oppressive intersections and effects of misfitting with normative temporalities. The thinking I share here is thus messy and unfinished, and I offer it up imperfectly in the hope it may grow and become otherwise.

## Mad time as a potential disruptor

I turn now to provide three examples of how Mad Time might be productive in disrupting normative and sanist research practices: rethinking data, embracing stumbling, circling, scrambling (becoming), and valuing stopping, starting, slowing down, and speeding up. I begin each example with a provocation from discussions with peer support workers where we talked about entanglements of time, mental health systems and/or experiences of distress or madness. I use these as a springboard to consider temporal violence and how such effects are potentially replicated or unsettled within methodological practice, offering my own experiences as a point of reflection or difference.

### Rethinking data

*“The clinicians are only seeing people at that stage. There’s a whole life out there, there’s a whole other story, and a whole other dimension to what’s going on for that person”* (Rebecca)

In our discussion, Rebecca and I talked about how clinicians often take a snapshot of one’s life, slicing and cutting time, assuming this is representative of one’s life. Mental health interventions are also often framed around the cutting of time: before diagnosis and treatment and after, when one is deemed recovered and, therefore, deemed suitable to return to societal roles. Time outside of intervention is rarely imagined or comprehended (Clare, 2017; Kafer, 2013).

This concept of temporal cutting can similarly be considered within the conceptualization and use of ‘data’. Data collection and analysis are commonly enacted as a linear, temporally bound process, where what counts as data is defined around a cutting of time. This occurs before ethics is approved, research proposals are written, and data is collected. Events or affective forces before (and sometimes even during) are unspoken of, hidden from view. Similarly, data is collected and *then* analyzed. Such a framing assumes a start-middle-end of research and knowledge that is discovered, captured, and isolated within such processes. Researchers are expected to think only about the data and nothing outside of the data.

Within such framings, the researcher is enacted as detached, following a strict methodological process to extract “truth nuggets from subjects,” uncontaminated by any influences that come before or during such data collection (Ellingson and Sotirin, 2020, p. 3). Individuals with experience navigating madness are predominantly produced as objects to either study or gain information from. Analysis happens after, belonging to the academic.

Within my own research, what might be referred to conventionally as the data set includes discussions with peer support workers, Australian mental health policy, and academic literature concerning peer support work in mental health systems. Following traditional conceptualizations of data, it is assumed that the knowledge produced is my own to claim, garnered from a rigorous independent analysis of temporally bound data.

Mad Time, however, invites us to blur such boundaries. Mad Time is messy and disruptive, blurring past, present, and future, subverting neat temporal boundaries around data. Morrigan (2017, p. 50) describes how, as someone living with complex trauma, they do not experience time as a “straightforward, orderly procession from the past, through the present, to the future.” Rather:

“the past rushes up on me with the urgency of the present. The future creeps out of crevices, leaking into the now. The future and past are intimately entwined, the present produced in their merging. Sections of time are uprooted and relocated” (Morrigan, 2017: 50).

Mad Time prompts a reconsideration of normative conceptualizations of data and, subsequently, who owns the knowledge produced. It invites us to speak to and incorporate, within research, the messy, overlapping nature of time and what occurs before, after and throughout data collection and analysis. To speak to, as Rebecca describes, the “whole other story” that’s going on outside of what’s traditionally considered data: the complex socio-material entanglements that produce knowledge. Thinking with Mad Time highlights that what we write about as research outcomes is not just from data as predominantly conceptualized. Rather, as researchers, we think from/through multiple experiences (both our own and others), concepts, subjective theories, desires, and material entanglements, folding across past–present–future, and it is these relations that produce knowledge.

For example, I research and write as a queer feminist, as having cared for disabled bodyminds, as a mother and community member, as having a lifetime of moving through (and not moving through) the world as someone who experiences periodic, debilitating distress, and as a facilitator of peer work training and supervision. I had been thinking and feeling through the implications of peer support inclusion within mental health systems for many years, with/in the community, before the formal structure of the research project. I have spoken with and listened to hundreds of individuals with experience of peer support, both before and during this research. My thinking and feeling about temporalities, madness and peer support happened whilst teaching; through attempting to crip time as part of my teaching practice alongside other instructors and theorizing with fellow peer workers during training sessions. It involved teaching my child, born during this research, about access needs and the importance of waiting so that we may all “move together” (Fritsch et al., 2021). It involved having my own expectations and understandings of time challenged by and through parenting, terminal illness, grief,^5^ madness and a pandemic. It happened with and through books, blogs, First Nation poetry, conversations at conferences, and social media posts. All of these are brought into and inform the research and yet extend beyond so-called data. The knowing and affect generated are thus collective, created through “relating criply and madly” (Eales and Peers, 2021, p. 164). It is these deeply entangled relations that produce knowledge.

In this way, thinking with Mad Time resonates with post-qualitative and Indigenous scholarship that argues for a consideration of data as embodied, relational and dynamic, assembled through the intra-action of individuals that contribute to the research, material objects, emotions, bodies, and cultural discourses within particular times and spaces (Ellingson and Sotirin, 2020; Koro-Ljungberg et al., 2017). It provokes a consideration of the deeply relational aspects of knowledge production and the potential of Mad individuals as critical theorists and activists. Whilst decentring the researcher as a sole agent, it invites us to consider our responsibility for our contributions, including how we are situated within relations that produce psy-logic and practices (Russo, 2022). It invites us to consider the way we make temporal cuts when conducting and describing methodology and what this means for the knowledge produced. As St Pierre (2014) prompts, what inquiry practices and concepts get left out? It asks us to consider whether data can ever be representative or whether it leaves out a “whole other dimension” (Rebecca). Bringing Mad Time into conversation with methodology thus has the potential to support further thinking, creatively and messily, about how we might conceptualize and use data differently in ways that do not simply replicate dominant ways of responding to madness.

### Embracing stumbling, circling, scrambling (becoming)

*“That was the circuitous path to becoming a peer worker” (Ben)*

In our discussion about peer support, Ben shared what he described as his “circuitous path” to becoming a peer support worker, one that resembled many of the journeys shared with me, my own included. Our paths to becoming peer workers felt convoluted, folding back and forth, and full of intervals. Such journeys are circuitous in comparison to the expectation that one progresses through normative life stages in a straight line – from past to present to a (curative) future (Kafer, 2013; Sheppard, 2020). Such expectations are particularly present for peer support workers, who are expected to recover and stay recovered (Sinclair, in press). Yet, such temporal framings do not reflect my own experiences, nor many of the other peer support workers I spoke with. My early employment history, for example, is littered with jobs abandoned due to an inability to maintain a consistent temporality of ‘climbing’ a career ladder, to drop down, return, start again, and move sideways.

Applying such “circuitous” orientations to research provokes a consideration of how traditional research practices are not only temporally bound but also chrono-normatively ordered. That is, methodological processes predominantly assume a linear, logical order from point A to point B, whereby the stages along the way are separable and distinct: from literature review to research question to data collection, analysis, and representation. This cleanness, linearity, predictability and consistency are considered the hallmarks of quality qualitative research (Koro-Ljungberg, 2016). Mad Time provokes a rethinking of such ordering. Rather than pathologize stumbling, circling and scrambling, Mad Time invites us to consider its potential value.

Looking at Koro-Ljungberg’s (2016) diagram of linearity in qualitative research processes, for example, I attempted to map my own methodological process between the discrete categories outlined in traditional methodological processes. My map looked like a higgledy-piggledy of back and forth, with so much folding between categories that the categories no longer made sense. What would traditionally be understood as data collection, for example, happened simultaneously as analysis. Different analytical questions emerged from plugging together various theories, feelings, experiences, methods, and thinking. As I thought and felt with others, the original research questions no longer fit, prompting me to circle back and reconsider the aims and tools needed (see Sinclair and Mahboub, 2024). So often, academic processes and expectations push us to continue moving forward, remaining fixed in our methodological orientations, only looking back as a reflection, not an intervention. And yet, had I stuck with the previous research questions and methods, I would have potentially replicated existing knowledge, refusing to listen to what was emerging from thinking and feeling collectively with/in the community.

Mad Time invites us to let go of what we thought the research would look like and what we thought it would become to allow for something new to emerge. Mad Time wrenches us out of what is expected. It allows for shifting ground for the unknown, giving us permission to take new paths to circle back when needed. We might understand that methods, affects, theories and analysis are not clearly defined by time but rather deeply entangled. We might think about the ways in which data may shape the researcher’s research question, prompt a new literature review, challenge, or create a new theory. Valuing these circuitous processes potentially enables something new to emerge within research that is less likely to replicate paths of psychiatry and other dominant frameworks of thinking and responding.

### Valuing variations in pace: stopping, starting, speeding up and slowing down

*“Everybody else is going on with their lives, and poor pathetic Chloe has to go and have a little hospital stay” (Chloe)*

As a final example, Mad Time provokes us to consider how we might shift research practices to not only accommodate but rather deeply value variations of pace. As Chloe articulates in the quote above, it is so often assumed that when we are experiencing madness, we must stop, exclude ourselves from life, and “disinvite myself from citizenship for a period of time,” as another peer support worker, Alex, described. Similarly, we are excluded from research because we fail to fit organizational time (Atkinson et al., 2024): schedules, priorities, pacing, and deadlines. However, sometimes, this is when our work may be the richest.

Doing research as someone who has a relationship with various forms of madness, the timing of my research practices is rarely smooth and consistent. My madness predominantly involves slowing down or stopping whilst the world seemingly races by without me. Moments, days, and weeks stretch out in a never-ending hopelessness. During these times, it would outwardly appear that my thinking and doing comes to a halt. Bruce (2021) describes such depressive time as lagging, dragging, lingering, and acting in slow motion, the value of which is unrecognizable to capitalist productivity. Sometimes time seems to stop whilst my mind goes around and around like a carousel with no way of me hopping off. Often, I will struggle to think in the morning, yet my mind will race at night. At other times, my madness fractures linear time: present and future disappear down a rabbit hole of past regrets, or I have a sense of impending doom that situates me squarely in the future, mostly paralyzing me but sometimes speeding me up in a flurry of nervous activity. In some of these moments, I can speed through and write large chunks of text, embracing a Mad Time that entails racing thoughts, restlessness, and hyperactivity.

Whilst usually only the latter speed is appreciated within academic research, when my bodymind slows, I can sink into research and experience a depth of thinking with theory. I do ‘analysis’, as suggested by St Pierre (2014) whilst living: whilst weeding the garden, showering, walking, and resting. Doing so enables a depth and breadth of thinking that is otherwise discouraged within an academic assemblage that values productivity and publications. Slowing down, for example, enables me to notice the knots in my stomach and the sense of unease produced through “hot spots” in the transcripts (Mac Lure, 2013, p. 172). Davies (2024) describes these as “maddening moments.” Instead of viewing these moments through a frame of pathology, we might wonder what these moments offer for research. I notice unease spread its tendrils into my everyday life, where I often see dominant beliefs and practices enacted throughout workplaces and personal relationships: psy-discourses, peer job description forms, mental health policy, peer support workers, and peer education. Embracing my own slowed temporality fosters a connection to the research. With the flexibility of choosing one’s own hours, working from home, using the Internet, and receiving support from family and friends, I have produced some of my best work during this ‘depressive time’. Rather than grinding toward a point of exhaustion for both our bodies and the planet, Price (2024, p. 78) highlights that “slowing down creates pauses and interstices that enable political theorizing, organizing, and intervention.”

Of course, there have been times where I have simply not been able to work because my bodymind has refused to move at all, insisting that I stop and rest. There are times when research may be enriched by stopping if needed or desired. There may also be times when we recognize the need or desire to move quickly when certain bodyminds desire or allow. We might consider the felt urgency of need: the way in which Mad thinkers are often told to slow down and be patient, that change takes time, whilst our peers continue to be harmed and neglected. Thinking with Mad Time is not about valuing a certain pace or temporality over another, but rather is about making space for multiplicity, honoring the productive nature of multiple speeds, temporal orientations, and desires within our research practices. It pushes us to consider how research practices might enable people to contribute at their own pace, knowing that doing so will enable not only epistemic justice but also deeper, richer research and activism.

One of the ways I did this in my own research was to provide materials in various formats ahead of time, allowing people to reflect/think/feel when and how it suited them. I invited individuals to contribute via various formats and did not restrict this to scheduled meetings, underpinned by a desire to unsettle the temporal disciplining regime of appointment time (Soldatic, 2013). How often within mental health assemblages must individuals be ready to recover when their appointment time with the worker is scheduled? Too often, one’s bodymind must adapt to the temporal rhythms of neoliberal systems. I tried to madden time by flexing to meet the needs of those I was meeting-morning, evening, breaks, talking slow, talking fast, and rescheduling. Other ways in which research might center Mad Time is through embracing asynchronicity and hybrid technologies, dedicating additional time to collective work, and enabling people to immerse themselves in flexible ways over the course of the research, as is often proposed for crip bodyminds (Atkinson et al., 2024).

Piepzna-Samarasinha (2022), for example, describes how embracing crip time means letting go of “abled panic” when technology fails, when certain deadlines are missed, or when someone’s access needs change. Rather than “giving up because the process is inefficient, non-standard, or slow,” we think outside the box, draw on other resources, and get creative (Gauthier-Mamaril, 2024, p. 1). As Gauthier-Mamaril (2024, p. 1) describes, “we all become risk assessors and masters of project management for our own energy and pain tolerance reserves.” Mad Time, like crip time, is generative. Mad individuals often have fabulously creative strategies for navigating the uncertainty of Mad Time. For myself, I have learnt to prioritize certain tasks that require speed or quick thinking when it best suits my bodymind, knowing other times might be suited to deep reflection. I am learning to let go of able-bodied panic, valuing instead the varied speeds at which my own and others’ bodyminds work. In working with contributors and fellow researchers, Atkinson et al. (2024) described cripping time as “prioritizing flexibility around hospital appointments, taking time off sick, waiting for antibiotics and other medications to kick in, and managing sudden hospitalizations….we build in contingencies, use organizational technologies to share and document our work so someone else can jump in when needed.” We may consider how such alternatives also madden time.

Whilst I provide these examples, we must also recognize that as part of academia or services reliant on funding, our ability to work collaboratively, to produce knowledge collaboratively, and to think about how to do so is constrained by temporal norms. For example, Scholz et al. (2019) document how temporal resources available within academia constrain research that positions individuals with experience navigating distress and/or mental health services as equal partners in the conceptualization, design, and undertaking of research. Collaborative research requires relationship building, developing collective viewpoints, being able to think together, reading with and against theory and struggling collectively, and the resources to do so are often limited on the ground. Furthermore, the time required for individuals deemed Mad to contribute to research, including the time of emotional labor and theorizing, is rarely recognized or valued (Faulkner and Thompson, 2021; Papoulias and Callard, 2022; Ross et al., 2023). I continue to sit with my own ethical discomfort in failing to unsettle many of these sanist effects within my own processes of inquiry whilst recognizing that the work of maddening methodology and the academy is collective.

## Conclusion

I started this article with two provocations. First, conventional methodologies are often temporally violent, producing effects similar to those of psychiatric relations. Second, Mad Time may be generative in subverting methodology in ways that move us toward the abolition of carceral understandings and responses to madness and a future in which madness does not equate to mandatory cure or treatment. Thinking and feeling with Mad Time and methodology holds the potential for moving beyond simply including individuals deemed mad into normative research approaches, where madness is accommodated or tolerated. It encourages us to consider not just more time in research but rather challenges the underpinning normative and normalizing expectations of pace, scheduling, and linear logic embedded in sanist methodological concepts and practices. As a starting point, I have proposed just a few ways Mad Time may be generative: by prompting a rethinking of data, embracing stumbling, circling, scrambling (becoming), and valuing variations in pace. By leaning into Mad Time, we might reimagine what counts as research and how research is conducted in ways that produce richer, more epistemically just knowledge and in ways that have practical implications for changing the world in which we live.

Thinking with Mad Time has implications not only for academia but also for other sites where knowledge is produced: in classrooms, during peer support catch-ups, within advocacy groups, around the vending machine, or on the communal couch in inpatient units. In what ways are we already maddening time in these spaces? How might recognizing and valuing Mad Time change the way in which we gather as activists or practice activism? How might the concept of Mad Time enable us to respond differently to our own and others’ madness in our everyday practices? Whilst I have argued that Mad Time is generative in its potential to disrupt normative research politics and practices, I look forward to further exploration of its generative potential both within and outside of academia and other conventional research spaces.

## Data Availability

The datasets presented in this article are not readily available because of ethics requirements. Requests to access the datasets should be directed to aimee.sinclair@postgrad.curtin.edu.au.

## Footnotes

^1^I use the term ‘bodymind’ to disrupt colonial and psychiatric enactments of mind–body as separate (see Clare, 2017; Price, 2015).

^2^By Mad futurities, I refer to Fritsch’s (2016: 11)‘s work, imagining the “flourishing of critical practices of an elsewhere and elsewhen of disability,” whereby madness is valued in the present, rather than overcome or cured in the future.

^3^I list only a few references here, in addition to those I quote throughout this article, in the hope they provide a gateway to further exploration and reading. It is vital that we recognise that Mad theorising and activism happens within, as well as outside of, academia.

^4^See for example, Youngblood Jackson and Mazzei (2011) conceptualisation of the interview as an ‘already failed practice’.

^5^Kenny et al. (2019) speak to some of the ways in which lived experiences of bereavement challenge normative linear understandings of bereavement and ‘recovery.’
